# Stimulation of Hemolysis and Eryptosis by β-Caryophyllene Oxide

**DOI:** 10.3390/life13122299

**Published:** 2023-12-04

**Authors:** Sumiah A. Alghareeb, Mohammad A. Alfhili, Jawaher Alsughayyir

**Affiliations:** Department of Clinical Laboratory Sciences, College of Applied Medical Sciences, King Saud University, Riyadh 12372, Saudi Arabia; 442204700@student.ksu.edu.sa (S.A.A.); malfeehily@ksu.edu.sa (M.A.A.)

**Keywords:** β-caryophyllene oxide, hemolysis, eryptosis, calcium, oxidative stress

## Abstract

Background: Eryptosis stimulated by anticancer drugs can lead to anemia in patients. β-caryophyllene oxide (CPO) is an anticancer sesquiterpene present in various plants; however, its effect on the structure and function of human red blood cells (RBCs) remains unexplored. The aim of this study was to investigate the hemolytic and eryptotic activities and underlying molecular mechanisms of CPO in human RBCs. Methods: Cells were treated with 10–100 μM of CPO for 24 h at 37 °C, and hemolysis, LDH, AST, and AChE activities were photometrically assayed. Flow cytometry was employed to determine changes in cell volume from FSC, phosphatidylserine (PS) externalization by annexin-V-FITC, intracellular calcium by Fluo4/AM, and oxidative stress by 2′,7′-dichlorodihydrofluorescein diacetate (H_2_DCFDA). Cells were also cotreated with CPO and specific signaling inhibitors and antihemolytic agents. Furthermore, whole blood was exposed to CPO to assess its toxicity to other peripheral blood cells. Results: CPO induced concentration-responsive hemolysis with LDH and AST leakage, in addition to PS exposure, cell shrinkage, Ca^2+^ accumulation, oxidative stress, and reduced AChE activity. The toxicity of CPO was ameliorated by D4476, staurosporin, and necrosulfonamide. ATP and PEG 8000 protected the cells from hemolysis, while urea and isotonic sucrose had opposite effects. Conclusions: CPO stimulates hemolysis and eryptosis through energy depletion, Ca^2+^ buildup, oxidative stress, and the signaling mediators casein kinase 1α, protein kinase C, and mixed lineage kinase domain-like pseudokinase. Development of CPO as an anticancer therapeutic must be approached with prudence to mitigate adverse effects on RBCs using eryptosis inhibitors, Ca^2+^ channel blockers, and antioxidants.

## 1. Introduction

**Figure 1:**
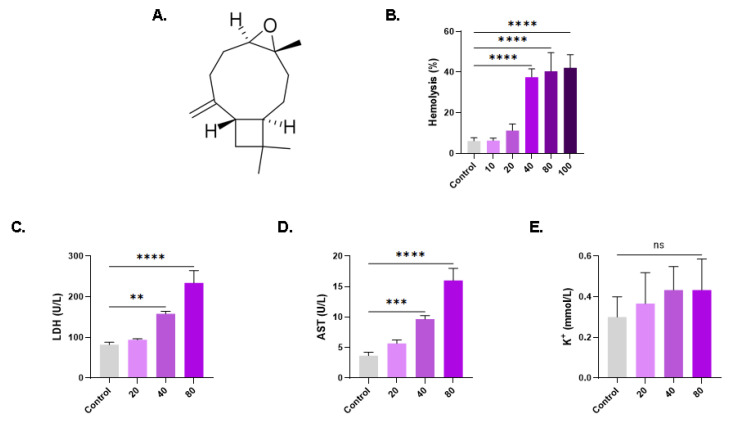
β-caryophyllene oxide induces hemolysis. (**A**) Molecular structure of CPO. (**B**) Dose-responsive hemolytic activity of CPO. Leakage of (**C**) LDH, (**D**) AST, and (**E**) K^+^ into the supernatant. ns indicates no significance, while ** (*p* < 0.01), *** (*p* < 0.001), and **** (*p* < 0.0001).

Chemical constituents derived from natural sources offer a compelling alternative for the formulation of anticancer pharmaceuticals, representing an economically viable therapeutic option [1]. β-caryophyllene oxide (CPO) is a sesquiterpene (Figure 1A) isolated from the essential oils of medicinal plants such as guava (*Psidium guajava*), cinnamon (*Cinnamomum* spp.), oregano (*Origanum vulgare* L.), black pepper (*Piper nigrum* L.), and clove (*Eugenia caryophyllata*). CPO has a wide range of pharmacological effects, including antibacterial, antifungal, immunomodulatory, anti-inflammatory, antirheumatic, and antioxidant properties [2]. Importantly, CPO has emerged as a promising anticancer compound because it primarily inhibits prostate, breast, and multiple myeloma cancer cell proliferation, angiogenesis, and metastasis by blocking Akt/mTOR/S6K1 and STAT3 signaling pathways and triggering apoptosis through reactive oxygen species (ROS) and MAPK activation [2,3]. Moreover, CPO stimulates apoptosis in lung cancer cells through mitochondrial stress, cell cycle arrest, and DNA fragmentation [4,5]. Altogether, current evidence argues for the great promise CPO holds as a prospective anticancer therapeutic.

Anemia, a major side effect of anticancer therapeutics encountered in at least 75% of patients, is attributed in part to myelosuppression, hemolysis, and eryptosis [6,7]. Eryptosis is a protective mechanism that eliminates aged and damaged red blood cells (RBCs) to prevent blood stasis, thrombosis, and uncontrolled hemolysis. However, when prematurely triggered by anticancer drugs, eryptosis leads to an excessive loss of circulating RBCs, and thus, to anemia [8]. Notably, eryptosis has also been identified as a contributing factor to numerous pathological conditions such as diabetes mellitus, renal failure, and anemia [9]. This mode of cell death is characterized by membrane blebbing, cell shrinkage, and phosphatidylserine (PS) exposure. Signaling enzymes involved in eryptosis include caspases, p38 MAPK, protein kinase C (PKC), casein kinase 1α (CK1α), mixed lineage kinase domain-like pseudokinase (MLKL), cyclooxygenase, and phospholipase A_2_ [10].

Numerous anticancer compounds, whether investigative or clinically approved, have been identified as triggers of hemolysis and eryptosis [11]. For example, paclitaxel, which causes anemia in patients, reportedly induces eryptosis through Ca^2+^ and ceramide accumulation and calpain stimulation [12]. Cisplatin is another chemotherapy that triggers Ca^2+^-dependent eryptosis through ATP exhaustion and loss of cellular volume [13]. Chemotherapy-related anemia is a serious and prevalent side effect of anticancer agents; therefore, it is prudent to assess the toxicity of investigative anticancer compounds to RBCs which offer an excellent cellular model for toxicological assessment [14,15]. Here, we explore the potential toxic effects of CPO on erythrocytes and elucidate the molecular mechanisms underlying these effects in an attempt to present a safety evaluation of CPO as an anticancer therapeutic.

## 2. Materials and Methods

### 2.1. Chemicals and Reagents

All chemicals were procured from Solarbio Life Science (Beijing, China). In total, 10 mg of CPO (CAS # 1139-30-6) was dissolved in 4.54 mL of dimethylsulfoxide (DMSO) to prepare a 10 mM stock solution stored as aliquots at –80 °C. Ringer solution was made up of 125 mM NaCl, 5 mM KCl, 1 mM MgSO_4_, 32 mM HEPES, 5 mM glucose, and 1 mM CaCl_2_, while phosphate-buffered saline (PBS) contained 137 mM NaCl, 2.7 mM KCl, 10 mM Na_2_HPO_4_, and 1.8 mM KH_2_PO_4_. KCl–Ringer was prepared by substituting NaCl and KCl for 125 mM KCl, and sucrose–Ringer contained 250 mM of sucrose instead of NaCl. Additionally, polyethylene glycol 8000 (PEG 8000) or urea were added to standard Ringer solution at 10% *w*/*v* and 300 mM, respectively [16].

### 2.2. Blood Collection

The protocol for this study was approved by the Ethics Committee of King Saud University Medical City (E-23-7764) and was conducted according to the Declaration of Helsinki. Blood samples were collected in lithium heparin or EDTA vacutainer tubes from 26 nonsmoking, healthy volunteers, 14 males and 12 females aged 24–35 years, with normal BMI and CBC results. RBCs were isolated by centrifugation (2500 RPM, 15 min, RT), washed twice in PBS, and suspended in either PBS or Ca^2+^-free Ringer solution at 4 °C for up to 24 h. Cells isolated from heparinized blood were treated with 10–100 μM of CPO for 24 h at 37 °C, while EDTA blood was diluted 1:2 in PBS and incubated with 80 μM of CPO for 24 h at 37 °C.

### 2.3. Hemolysis

Following treatment with CPO under different experimental conditions, the supernatants of control and treated cells were harvested by centrifugation at 13,000× *g* for 1 min at RT. Light absorbance at 405 nm was recorded using an LMPR-A14 microplate reader (Labtron Equipment Ltd., Surrey, UK); RBCs suspended in ddH_2_O served as a positive control [17]. The following equation was used to derive percentage hemolysis:

(1) $$Hemolysis\;\left(\%\right)=\frac{\left(OD\;blank\right)-(OD\;treated)}{\left(OD\;blank\right)-(OD\;positive)}\times 100$$

### 2.4. Potassium Leakage

The K^+^ content of the supernatants was measured using a Blood Potassium Content Assay Kit (Solarbio). In brief, K^+^ in the supernatants of control and treated cells reacts with sodium tetraphenylborate to form insoluble K^+^ tetraphenylborate whose turbidity (λ_max_ = 520 nm) is proportional to the concentration of K^+^ in the sample [18].

### 2.5. Lactate Dehydrogenase (LDH) Activity

Solarbio’s LDH Activity Assay Kit was used to measure LDH activity in the supernatants. In the reaction, LDH reduces NAD^+^ to NADH and oxidizes lactate to pyruvate, which then reacts with 2,4 dinitrophenylhydrazine to produce pyruvate dinitrobenzene, whose brown-red color under alkaline conditions is proportional to its pyruvate content (λ_max_ = 450 nm). One unit of enzyme activity is defined as the amount of enzyme necessary to produce 1 nM of pyruvate per min per ml supernatant [19].

### 2.6. Aspartate Aminotransferase (AST) Activity

Solarbio’s AST Activity Assay Kit was used to measure AST activity. AST transfers an amine group from aspartate to α-ketoglutarate, which, in turn, generates glutamate and oxaloacetate. The resulting oxaloacetate is decarboxylated to pyruvate, which reacts with 2,4-dinitrophenylhydrazine to produce brownish-red 2,4-dinitrophenylhydrazone under alkaline conditions (λ_max_ = 505 nm) proportional in intensity to AST activity [20]. One unit of enzyme activity is the amount of enzyme necessary to produce 1 μM of pyruvate per min per ml hemolysate.

### 2.7. Osmotic Fragility

Cells were suspended in an isotonic solution (0.9% NaCl) for 1 h at 37 °C in addition to hypotonic solutions of 0.7% NaCl, 0.5% NaCl, 0.3% NaCl, and 0.1% NaCl, and in distilled water (i.e., 0% NaCl) with and without sub-hemolytic concentrations of CPO (1, 5, 10, and 20 µM) [18]. Hemolysis was then determined as described earlier.

### 2.8. Cell Signaling Analysis

Hemolysis was measured in RBCs exposed for 24 h at 37 °C to the vehicle (0.1% DMSO) or to 40 µM of CPO with or without p38 MAPK inhibitor (SB203580; 100 µM), CK1α inhibitor (D4476; 20 µM), PKC inhibitor staurosporin (StSp; 1 µM), MLKL inhibitor necrosulfonamide (NSA; 0.5 µM), Rac GTPase inhibitor (NSC23766; 100 µM), melatonin (MTN; 5 µM), ATP (500 µM), Ca^2+^ chelator glycine, N,N′-[1,2-ethanediylbis(oxy-2,1-phenylene)]bis[N-[2-[(acetyloxy)methoxy]-2-oxoethyl]]-, bis[(acetyloxy)methyl] ester (BAPTA-AM; 10 µM), vitamin C (1 mM), nitric oxide synthase inhibitor (L-NAME; 20 µM), or cyclooxygenase inhibitor acetylsalicylic acid (ASA; 25 µM).

### 2.9. Membrane Scrambling

Annexin-V-FITC was used to stain control and treated cells (20, 40, and 80 μM). Aliquots of cell suspensions were incubated with 1% annexin-V-FITC for 10 min at RT in the dark, and the fluorescence was recorded at Ex/Em of 488/512 nm using a Northern Lights flow cytometer (Cytek Biosciences, Fremont, CA, USA) [21].

### 2.10. Cellular Morphology

Cell size and granularity of control and treated (20, 40, and 80 μM) cells were inferred from forward scatter (FSC) and side scatter (SSC) signals, respectively. Additionally, micrographs of control and treated (80 μM) RBCs were obtained following Giemsa staining (Atlas Medical, Cambridge, UK) [22].

### 2.11. Erythrocyte Sedimentation Rate (ESR)

The sedimentation rates of RBCs in control and treated whole blood (80 μM) were measured in Westergren tubes [23]. Following treatment, Westergren tubes were loaded with whole blood and incubated for 1 h at RT away from light to allow cells to vertically sediment; the distance travelled (mm/h) was then recorded.

### 2.12. Acetylcholine Esterase (AChE) Activity

The catalytic activity of AchE was assayed using Solarbio’s AchE Activity Assay Kit, based on Ellman’s method. Hemolysates of control and treated cells (40, 80, and 100 μM) were reacted with acetylthiocholine to form thiocholine which, upon reacting with 2-nitrobenzoic acid, generates 5-mercaptonitrobenzoic acid, whose absorbance at 412 nm is proportional to thiocholine, and hence, to AChE activity [24]. One unit of enzyme activity is defined as the amount of enzyme that catalyzes the generation of 1 nM of 5-mercaptonitrobenzoic acid per min per ml hemolysate.

### 2.13. Extracellular Acidity

The pH values of the supernatants of control and treated RBCs (20, 40, and 80 μM) were determined via ion-selective electrode using the EXIAS e|1 analyzer (EXIAS Medical GmbH, Graz, Austria) [25].

### 2.14. Intracellular Ca^2+^

Fluo4/AM is a cell-permeable Ca^2+^ probe used to measure intracellular Ca^2+^ contents. In brief, control and treated cells (20, 40, and 80 μM) were incubated with 2 μM of Fluo4/AM for 30 min at 37 °C in the dark. Cells were then washed twice to remove excess and unbound stain, and the green Fluo4 fluorescence was finally captured by flow cytometry at 512 nm following excitation by the blue laser at 488 nm [26].

### 2.15. Oxidative Stress

ROS in control and treated cells (20, 40, and 80 μM) were stained with 2′,7′-dichlorodihydrofluorescein diacetate (H_2_DCFDA). Cells were incubated with 10 μM of H_2_DCFDA for 30 min at 37 °C away from light and washed twice to remove excess dye. Inside cells, H_2_DCFDA is hydrolyzed by esterases into DCF which is excited by the blue laser at 488 nm to emit green fluorescence at 512 nm detected by flow cytometry [27].

### 2.16. Systemic Toxicity

A complete blood count test was performed on control and treated whole blood (80 μM) using the BC-6200 hematology analyzer (Mindray Medical International Limited, Shenzhen, China), as previously described [28].

### 2.17. Statistical Analysis

The results are presented as means *±* SD of three independent experiments, with each run performed in triplicate (*n* = 9). Control and treated cells from the same donor were used in distinct experiments to account for individual variations in cell susceptibility. FlowJo™ v10.7.2 (Becton, Dickinson and Company, Ashland, OR, USA) was used to analyze flow cytometry data and all statistical tests were performed in GraphPad v9.5.1 (GraphPad Software, Inc., San Diego, CA, USA). Student’s *t*-test was used to compare two groups, whereas one-way ANOVA followed by Dunnett’s test was used to compare three or more groups. The results were judged to be statistically significant at *p* < 0.05.

## 3. Results

### 3.1. CPO Induces Concentration-Dependent Hemolysis

To determine whether CPO is cytotoxic to RBCs, hemolysis was assessed. In comparison to the control group (6.16% *±* 1.56%), CPO induced significant hemolysis at 40 µM (37.57% ± 4.05%, *p* < 0.0001), 80 µM (40.42% ± 9.22%, *p* < 0.0001), and 100 µM (42.15% ± 6.43%, *p* < 0.0001), as shown in Figure 1B. We also identified hemolytic markers targeted by CPO, and, accordingly, erythrocytes treated with 40 µM and 80 µM of CPO exhibited a significant release of LDH (Figure 1C) and AST (Figure 1D) but not K^+^ (Figure 1E).

### 3.2. CPO Does Not Protect against Hypotonic Hemolysis

**Figure 2:**
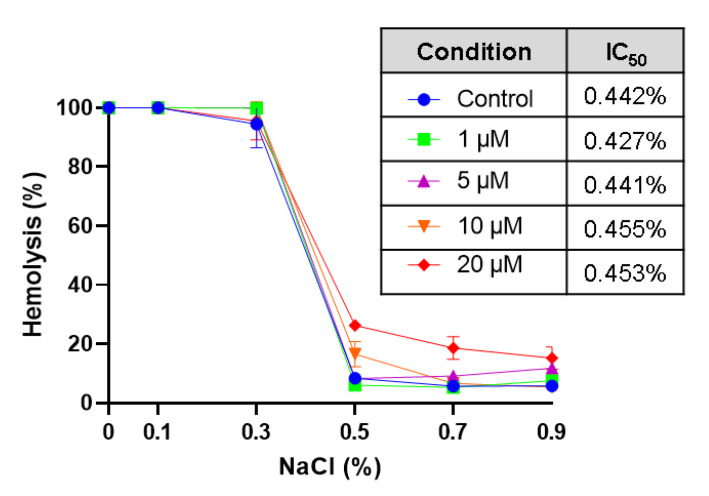
Effect of CPO on osmotic fragility. Cells were incubated in isotonic and hypotonic NaCl solutions with and without 1–20 μM of CPO for 1 h at 37 °C; hemolysis was then determined.

In order to investigate whether sub-hemolytic concentrations of CPO (1, 5, 10, and 20 µM) offer mechanical stability against hypotonic hemolysis, we conducted osmotic fragility tests. As shown in Figure 2, CPO had no protective effect against the hypotonic lysis of RBCs.

### 3.3. CPO-Induced Hemolysis Is Mediated through Multiple Signaling Pathways

**Figure 3:**
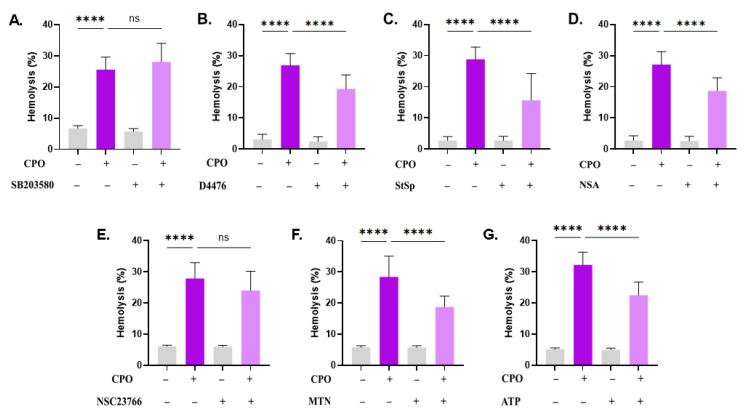
CPO-induced cell death is mediated through several pathways. Effect of (**A**) SB (100 µM), (**B**) D4476 (20 µM), (**C**) STSP (1 µM), (**D**) NSA (500 nM), (**E**) NSC (100 µM), (**F**) MTN (5 µM), and (**G**) ATP (500 µM) on the hemolytic activity of CPO (40 μM). ns indicates no statistical significance, while **** (*p* < 0.0001).

Next, it was of interest to probe the signal transduction pathways involved in CPO-induced hemolysis using small molecule inhibitors. The presence of SB203580, ASA, and NSC23766 did not significantly inhibit CPO-mediated hemolysis (Figure 3A,E,F). Nevertheless, hemolysis was significantly decreased upon treatment with D4476 (26.9% ± 3.84% vs. 19.34% ± 4.4%, *p* < 0.0001, Figure 3B), StSp (28.82% ± 3.95% vs. 15.7% ± 8.53%, *p* < 0.0001, Figure 3C), NSA (27.17% ± 4.13% vs. 18.72% ± 4.20%, *p* < 0.0001, Figure 3D), MTN (28.4% ± 6.64% vs. 18.76% ± 3.45%, *p* < 0.0001, Figure 3F), and ATP (32.26% ± 4.04% vs. 22.51% ± 4.25%, *p* < 0.0001, Figure 3G).

### 3.4. CPO Causes Cell Shrinkage and Disrupted Morphology

**Figure 4:**
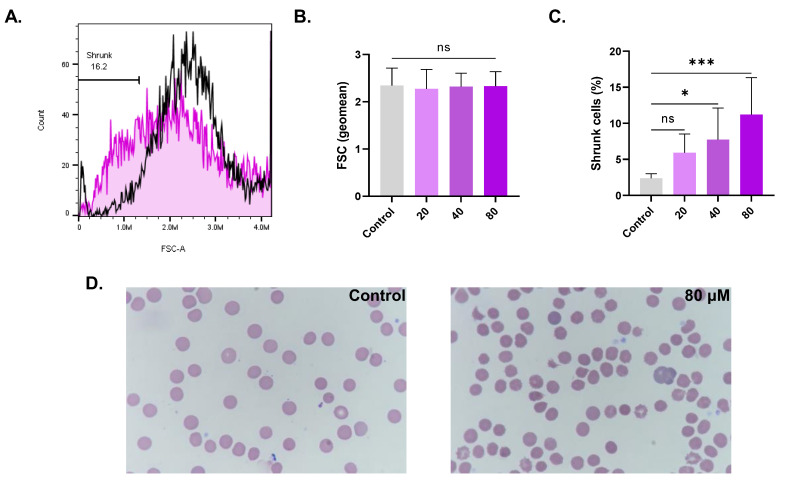
Effect of CPO on RBC morphology. (**A**) Representative histograms of the FSC distribution of control (black line) and treated (pink line, 80 μM) cells. (**B**) Geomean of FSC. (**C**) Percentage of shrunk cells. (**D**) Bright-field micrographs of Giemsa-stained control and treated RBCs (100x). ns indicates no statistical significance, while * (*p* < 0.05) and *** (*p* < 0.001).

Hemolysis and eryptosis are associated with cell shrinkage and morphological abnormalities. As evident in Figure 4C, the percentage of cells undergoing shrinkage significantly increased from control values of 2.38% ± 0.63% to 7.73% ± 4.37% (*p* < 0.05) at 40 µM and to 11.20% ± 5.11% (*p* < 0.001) at 80 µM. Moreover, morphological changes including schistocytes and acanthocytes were observed following the Giemsa staining of control and treated (80 µM) RBCs (Figure 4D).

### 3.5. CPO Triggers PS Externalization

**Figure 5:**
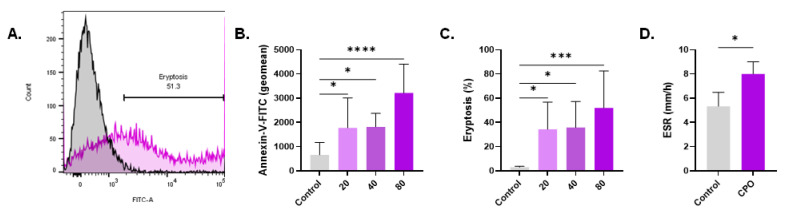
CPO elicits eryptosis. (**A**) Representative histograms of the distribution of control (black line) and treated (pink line, 80 μM) relative to annexin-V-FITC fluorescence. (**B**) Geomean of Annexin-V-FITC fluorescence. (**C**) Percentage of eryptotic cells. (**D**) ESR of control and treated (80 μM) cells in whole blood. * (*p* < 0.05), *** (*p* < 0.001), and **** (*p* < 0.0001).

Translocation of PS is a hallmark of eryptosis. As shown in Figure 5B, CPO-treated cells demonstrated a concentration-dependent increase in the geometric mean of annexin-V-FITC fluorescence from 662.1 ± 510.4 arbitrary units (a.u.) to 1779 a.u. ± 1230 a.u. (20 µM, *p* = 0.0461), 1818 a.u. ± 555.9 a.u. (40 µM, *p* = 0.0450), and 3213 a.u. ± 1187 a.u. (80 µM, *p* < 0.0001). Similarly, the percentage of cells exhibiting PS exposure demonstrated a consistent pattern of increase from 3.02% ± 0.75% to 34.37% ± 22.45% (20 µM, *p* = 0.0248), 35.76% ± 21.56% (40 µM, *p* = 0.0185), and 52.06% ± 30.39% (80 µM, *p* = 0.0005), as shown in Figure 5C. Eryptotic cells aggregate more strongly than normal cells. In alignment with PS exposure, the ESR of CPO-treated cells (80 µM) demonstrated a significant increase compared with control cells (5.33 mm/h ± 1.16 mm/h to 8.0 mm/h ± 1.0 mm/h, *p* = 0.0390, Figure 5D).

### 3.6. Diminished AChE Activity and pH in Response to CPO

**Figure 6:**
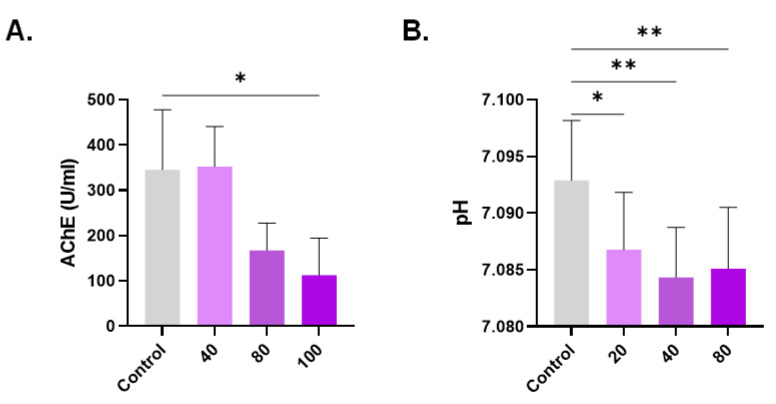
Anticholinesterase activity of CPO. (**A**) Intracellular AChE activity and (**B**) supernatant pH. * (*p* < 0.05) and ** (*p* < 0.01).

AChE activity is diminished in aged and damaged cells; therefore, we determined the anticholinesterase potential of CPO. AChE activity was significantly reduced from 345.80 U/L ± 132 U/L to 112.8 U/L ± 82.14 U/L upon the exposure of cells to 100 µM of CPO (Figure 6A). Additionally, increased extracellular acidity (Figure 6B) was noted as the drop in pH from 7.093 ± 0.01 was statistically significant at 20 μM (7.087 ± 0.01, *p* < 0.05), 40 μM (7.084 ± 0.004, *p* < 0.01), and 80 μM (7.085 ± 0.005, *p* < 0.01).

### 3.7. CPO Raises Cytosolic Ca^2+^

**Figure 7:**
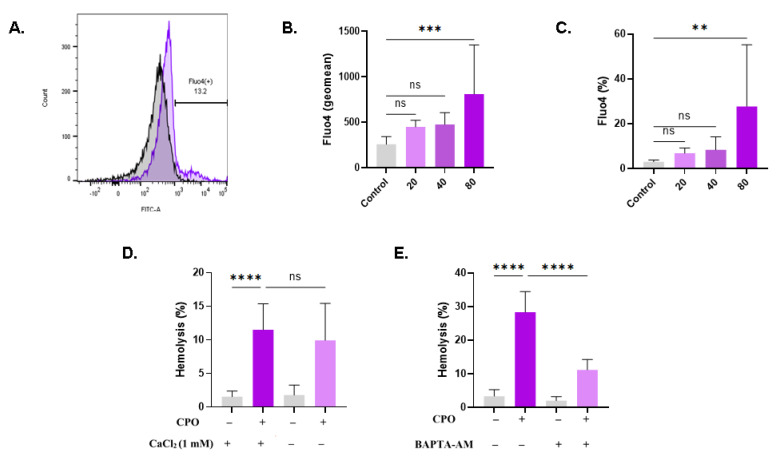
CPO increases cytosolic Ca^2+^ levels. (**A**) Representative histograms of Fluo4 fluorescence in control (black line) and treated (pink line, 80 μM) cells. (**B**) Geomean of Fluo4 fluorescence. (**C**) Percentage of cells with Ca^2+^ accumulation. (**D**) Effect of extracellular Ca^2+^ elimination on hemolysis. (**E**) Inhibition of hemolysis by 10 μM of BAPTA-AM. ns indicates no statistical significance, while ** (*p* < 0.01), *** (*p* < 0.001), and **** (*p* < 0.0001).

The elevation of intracellular Ca^2+^ is the main mechanism underlying hemolysis and eryptosis. CPO only resulted in a significant increase in Fluo4 fluorescence at 80 µM (258.3 a.u. ± 85.40 a.u. to 810.3 a.u. ± 539.5 a.u., *p* = 0.0007), as depicted in Figure 7B. In agreement with this finding, the percentage of RBCs exhibiting increased Ca^2+^ was significantly higher in treated cells (27.73% ± 27.53% at 80 µM) compared with control values of 3.04% ± 0.76% (Figure 7C). Notably, hemolysis was not affected by extracellular Ca^2+^ removal (Figure 7D), whereas the addition of BAPTA-AM (Figure 7E) rescued the cells from hemolysis (28.36% ± 6.08% to 11.19% ± 3.06%, *p* < 0.0001).

### 3.8. CPO Elicits Oxidative Stress

**Figure 8:**
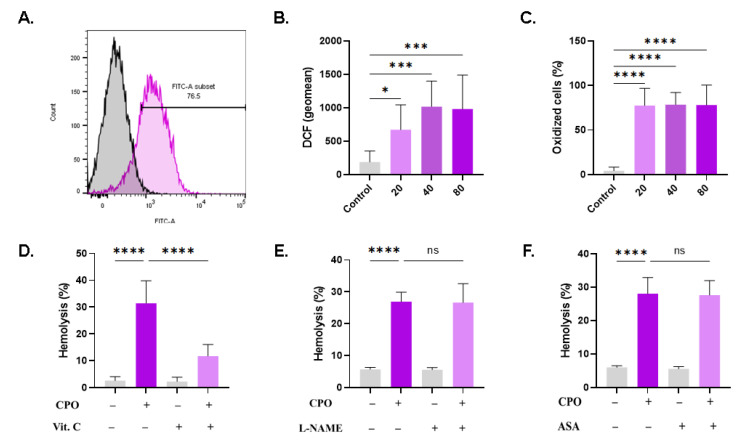
CPO promotes oxidative stress. (**A**) Representative histograms of DCF fluorescence in control (black line) and treated (pink line, 80 μM) cells. (**B**) Geomean of DCF fluorescence. (**C**) Percentage of oxidized cells. Effect of (**D**) 1 mM of vitamin C, (**E**) 20 μM of L-NAME, and (**F**) 25 µM of ASA on hemolysis. ns indicates no statistical significance, while * (*p* < 0.05), *** (*p* < 0.001), and **** (*p* < 0.0001).

Hemolysis and eryptosis are associated with redox imbalance and the accumulation of ROS. CPO-treated RBCs exhibited a significant and concentration-dependent increased in DCF geomean from 187.0 a.u. ± 165.5 a.u. to 671.5 a.u. ± 372.7 a.u. (20 µM, *p* = 0.0363), 1017 a.u. ± 381.2 a.u. (40 µM, *p* = 0.0003) and 980.4 a.u. ± 509.3 a.u. (80 µM, *p* = 0.0005), as shown in Figure 8B. Additionally, the percentage of oxidatively stressed cells was significantly increased (Figure 8C). ROS are neutralized by antioxidants; therefore, it was of interest to investigate the potential protective effect offered by vitamin C against CPO toxicity. Additionally, given the crosstalk between oxidative stress and both nitric oxide synthase and cyclooxygenase, we tested whether the inhibition of either enzyme could ameliorate CPO-induced hemolysis. Notably, the hemolytic activity of CPO was significantly reduced upon incubating the cells with vitamin C (31.47% ± 8.39% to 11.76% ± 4.34%, *p* < 0.0001; Figure 8D), but not with L-NAME (Figure 8E) or ASA (Figure 8F).

### 3.9. Effect of Modified Ringer Solutions on CPO Toxicity

**Figure 9:**
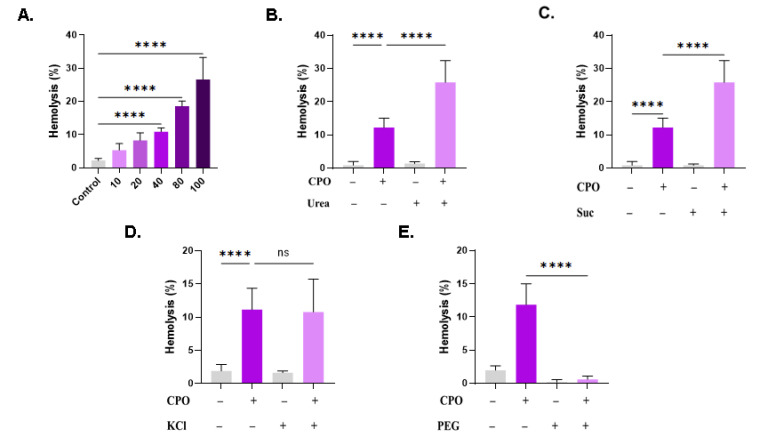
Effect of modified Ringer solutions on CPO toxicity. (**A**) Dose-responsive hemolytic activity of CPO in standard Ringer solution. Effect of (**B**) urea (300 mM), (**C**) sucrose (250 mM), (**D**) KCl (125 mM), and (**E**) PEG (10% *w*/*v*) on CPO-induced hemolysis (40 μM). ns indicates no statistical significance, while **** (*p* < 0.0001).

Next, we examined the potential effect of extracellular space modifications on CPO-induced hemolysis. In particular, isotonic urea and sucrose may blunt or aggravate cytotoxicity depending on their interaction with the hemolytic compound. The hemolytic effect of CPO suspended in standard Ringer solution reached statistically significant levels at 40 µM (10.88% ± 1.01%, *p* < 0.0001), 80 µM (18.56% ± 1.55%, *p* < 0.0001), and 100 µM (26.64% ± 6.61%, *p* < 0.0001), as evident from Figure 9A. The addition of urea significantly augmented the hemolytic effect of CPO (8.12% ± 3.70% vs. 28.76% ± 5.17%, *p* < 0.0001, Figure 9B), as did sucrose (12.19% ± 2.83% vs. 25.82% ± 6.56%, *p* < 0.0001, Figure 9C). The presence of extracellular KCl at 125 mM blocks KCl exit from the cells. However, this manipulation failed to offer significant protection from hemolysis (Figure 9D). PEG compounds physically coat cells and compounds, which may therefore attenuate their toxicity. As shown in Figure 9E, PEG 8000 thoroughly abrogated the hemolytic activity of CPO (11.82% ± 3.19% to 0.58% ± 0.49%, *p* < 0.0001).

### 3.10. CPO Causes Distinct Alterations in Whole Blood

**Figure 10:**
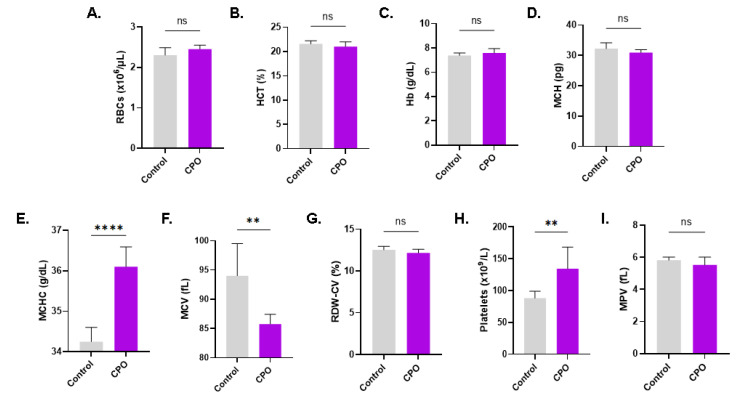
Effect of CPO on whole blood: (**A**) RBC count, (**B**) hematocrit (HCT), (**C**) Hb, (**D**) mean corpuscular Hb (MCH), (**E**) MCHC, (**F**) MCV, (**G**) red cell distribution width-coefficient of variation (RDW-CV), (**H**) platelet count, and (**I**) mean platelet volume (MPV) of control and CPO-treated (80 μM) whole blood. Ns indicates no statistical significance, while ** (*p* < 0.01) and **** (*p* < 0.0001).

**Figure 11:**
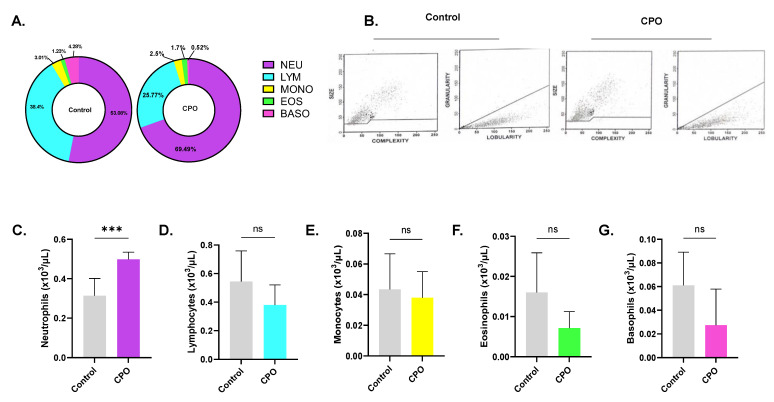
Effect of CPO on white blood cell subsets. (**A**) Differential count of leukocytes. (**B**) Representative dot plots of size, complexity, and lobularity of leukocytes. Viability of (**C**) neutrophils, (**D**) lymphocytes, (**E**) monocytes, (**F**) eosinophils, and (**G**) basophils. ns indicates no statistical significance, while *** (*p* < 0.001).

Finally, we investigated the toxicity of CPO to other peripheral blood cells. No significant changes in RBC indices were noted after CPO treatment (Figure 10), except for the mean corpuscular Hb concentration (MCHC), which significantly increased from 34.25 g/dL ± 0.35 g/dL to 36.10 ± 0.49 g/dL (*p* < 0.0001), and the mean corpuscular volume (MCV), which was significantly reduced from 94.02 fL ± 5.49 fL to 85.77 fL ± 1.66 fL (*p* = 0.0055), as shown in Figure 10E,F, respectively. CPO also seemed to have a stimulatory effect on platelet maturation, resulting in a substantial rise in platelet count (88.13 × 10^9^/L ± 10.77 × 10^9^/L to 134.4 × 10^9^/L ± 33.94 × 10^9^/L Figure 10H) without affecting platelet volume (Figure 10I). Furthermore, as shown in Figure 11C, the percentage of neutrophils significantly increased from 48.94% ± 0.18% to 62.07% ± 0.29% upon CPO treatment (*p* = 0.0028). On the other hand, the percentages of the other leukocyte subsets were reduced upon CPO treatment, but did not reach statistical significance (Figure 11D–G). CPO is therefore not toxic to white blood cells.

## 4. Discussion

CPO has been shown to exert a broad spectrum of pharmacological effects, not only through inhibiting the proliferation of various tumor cell types, but also by being an antimicrobial, anti-inflammatory, and an antirheumatic agent [2] that targets a vast array of intracellular networks. One of the essential characteristics of potential cancer therapeutics is the lack of toxicity to off-target tissue, most notably RBCs, to circumvent the complexities that may arise due to chemotherapy-associated anemia. Given that eryptosis impedes the transport of oxygen and is a recognized factor of anemia, the novel findings presented herein inform and guide the future development of CPO as an anticancer agent.

This study has disclosed, for the first time, that anticancer concentrations of CPO (30–100 μM) [2,4,5] possess hemolytic (Figure 1) and eryptotic (Figure 5) properties with morphological alterations including anisocytosis, schistocytosis, and acanthocytosis (Figure 4). The hydrophobic nature of CPO allows it to bind and disrupt the RBC lipid bilayer, leading to increased membrane permeability and the subsequent efflux of cellular components [29]. Once eryptosis is triggered, intracellular levels of Ca^2+^, ROS, and ceramide increase. The increase in Ca^2+^ ions consequently leads to KCl and water efflux and eventual shrinkage [30]. Thus, cell shrinkage (Figure 4) is secondary to the intracellular buildup of Ca^2+^ (Figure 7), which opens Ca^2+^-responsive K^+^ channels, leading to KCl and water exit. Interestingly, the hemolytic potential of CPO was effectively suppressed when co-administered with the cell-permeable Ca^2+^-chelating agent BAPTA-AM, further substantiating the pivotal role of Ca^2+^ in the toxicity of CPO. Interestingly, senicapoc, a Gardos channel inhibitor that maintains ion trafficking and preserves volume homeostasis, has been investigated for sickle cell disease and hereditary xerocytosis, with promising findings [31,32].

The hemolytic activity of CPO seems to be mediated by multiple signaling pathways. CK1α is a protein kinase that belongs to the serine/threonine kinase family and has a crucial role in the regulation of various physiological processes, such as the apoptosis of nucleated cells [33]. CK1α can also regulate Ca^2+^ trafficking and membrane scrambling in response to energy deprivation [34]; thus, blocking CK1α activity may reduce the hemolytic effect of CPO on RBCs in vivo. Notably, several types of cancer exhibit a differential regulation of CK1α, which makes it a potential target for anticancer therapy. Thus, the use of CPO and CK1α inhibitors, most importantly D4476, deserves further examination given that it significantly prevents CPO toxicity to RBCs. BTX-A51, another CK1α inhibitor, has reached clinical trials in a first-in-human study [35].

PKC inhibitor StSp also targets the PI3K/AKT pathway [36] and inhibits the proliferation of many human cancer cell lines [37]. In this study, StSp was identified as an inhibitor of CPO-mediated hemolysis (Figure 3), possibly implicating PKC or PI3K/AKT as putative targets of CPO. PKC is activated upon metabolic exhaustion [7] and ATP rescues cells from hemolysis (Figure 3); therefore, it follows that CPO may lead to energy depletion, which, in turn, activates PKC and leads to cell death. Similarly to CK1α, PKC isoforms exhibit heterogeneous expression profiles in various types of cancer, probably due to frequent mutations [38]. Experimental evidence indicates that PKC promotes cancer cell survival by activating a number of anti-apoptotic, autophagic, necroptotic, and ferroptotic mediators. As such, PKC inhibitors, such as StSp and midostaurin, constitute an attractive target for anticancer therapy. It has been demonstrated, for instance, that StSp sensitizes chronic myeloid leukemia cells to imatinib by arresting cell cycle progression [39]. Indeed, our results indicate that CPO-induced hemolysis is mediated through PKC (Figure 3), whose blockade may therefore augment the sensitivity of cancer cells to CPO while simultaneously protecting RBCs.

The generation of ATP within RBCs is of great significance due to its crucial role in supporting the energy-dependent regulation of ionic and structural equilibrium within RBCs, which are subject to mechanical and chemical pressure throughout the microvasculature [40]. ATP depletion increases the vulnerability of RBCs to injury and hemolysis; examination of the effect of CPO on cellular energetics, most notably glycolytic enzymes and lactate accumulation, is, therefore, of interest to future studies. It is important to mention that increased extracellular acidity was noted in treated cells (Figure 6), which suggests lactic acid buildup.

Necroptosis has been observed in RBCs in response to pore-forming bacterial toxins and to cryostorage [41,42], and the necroptotic executioner, MLKL, is recruited and phosphorylated by PI3K, which leads to cell membrane perforation [43]. Cancer cells may develop resistance to apoptosis; thus, targeting other modalities of cell death has recently gained considerable attention. In fact, the suppression of MLKL is important for cancer cell survival and metastasis [44]. Therefore, a growing number of compounds have been identified as necroptosis inducers with great potential for therapeutic development. Conversely, MLKL activation has been reported to participate in insulin resistance, demyelination, and hepatocellular injury [45]. Our results demonstrate the protective role of NSA against hemolysis, suggesting the involvement of the RIPK1/RIPK3/MLKL pathway in CPO-induced RBC death. Along those lines, NSA alleviates acute brain injury in intracerebral hemorrhage [46], intestinal inflammation [47], and pulmonary ischemia–reperfusion injury [48]. Thus, mounting evidence argues for the use of NSA as an ancillary adjuvant alongside CPO to reduce its toxicity to erythrocytes. Nevertheless, if and how the MLKL blockade through NSA interferes with the anticancer potential of CPO remains to be determined.

The compromised structural integrity of the membrane of eryptotic cells (Figure 5) causes RBCs to adhere to the endothelial lumen, which is further substantiated by the elevated ESR, indicative of enhanced blood viscosity, which significantly predisposes patients to thromboembolic events [49]. Notably, eryptosis is associated with reduced AChE activity (Figure 6). AChE is a glycosylphosphatidylinositol-linked membranous enzyme that helps maintain the shape and size of RBCs and is used as a marker of cellular aging and membrane integrity [50]. It is implied that CPO possesses an anticholinesterase activity which may underlie the morphological changes caused by sesquiterpene (Figure 4) [51].

We have also identified oxidative stress as a central mechanism in CPO toxicity to RBCs (Figure 8). The presence of MTN (Figure 3) or vitamin C (Figure 6) partially mitigated CPO toxicity, indicating that ROS formation is essential to the hemolytic activity of CPO. Notably, vitamin C induces ROS generation and PKC activation, which elevates cytosolic Ca^2+^, leading to necrosis in laryngeal squamous cell carcinoma [52]. Thus, it seems reasonable to assume that ROS and PKC are intricately linked in mediating CPO toxicity in RBCs. MTN is an antioxidant [53] and an iron chelator [54] that also interacts with calmodulin: a protein involved in the regulation of Ca^2+^. Therefore, the decrease in hemolysis observed in the presence of MTN suggests that it may also be related to Ca^2+^ signaling (Figure 7). In fact, several reports have demonstrated the antitumor properties of MTN which, at least in part, are attributed to its antioxidant activity [55]. Since oxidative stress promotes tumor growth, antioxidants, as demonstrated by our current findings, may maximize the anticancer potential of CPO while rescuing RBCs from hemolysis and eryptosis.

Our results indicate that both urea and sucrose aggravate the hemolytic potential of CPO (Figure 9). While the protective role of urea to RBCs has been described [56], urea, being a uremic toxin, has also been reported to reduce the RBC lifespan [57]. However, the mechanisms underlying both effects remain poorly understood. It is reasonable to assume that the presence of CPO blocked the antihemolytic activity of urea by interacting with a shared molecular target. This target may be sphingomyelinase, the Na^+^-K^+^-ATPase pump, the KCl cotransporter, or the Na^+^-K^+^-2Cl^−^ cotransporter [56]. Future studies should examine the possible effect of CPO on these targets in the presence and absence of urea. Moreover, because sucrose is of relatively low dielectric constant, the finding that it augmented the hemolytic activity of CPO suggests that the interaction of CPO with erythrocytes is primarily ionic in nature. Similarly, CPO may antagonize processes targeted by sucrose to prevent hemolysis, such as limiting water entry and Cl^–^ efflux [58]. Conversely, the most potent inhibitor of CPO toxicity to RBCs identified in this study was PEG 8000 (Figure 9). This observation indicates that cells have been physically protected from CPO either by entrapment of the compound in PEG molecules and/or by being themselves ensnared within. The antitumor activity, bioavailability, and toxicity profile of PEGylated CPO preparations must thus be investigated in vivo.

Our study is not without limitations. First, physiological responses of cells grown in culture do not necessarily mirror those encountered in vivo. Second, the effect of CPO on oxidative stress markers and energy turnover is lacking. Third, our study does not facilitate the determination of CPO metabolism and bioavailability.

In conclusion, the current study reveals novel hemolytic and eryptotic activities of CPO characterized by physical damage of the cell membrane, phospholipid scrambling, morphological changes, Ca^2+^ accumulation, loss of cellular volume, oxidative stress, AChE inhibition, and metabolic exhaustion. These manifestations are mediated through multiple signaling pathways, including CK1α, PKC, and MLKL, whose inhibition may reverse the toxic effects of CPO to erythrocytes. Thus, in light of contemporary evidence, the development of CPO as an anticancer therapeutic must be cautiously considered in addition to the use of eryptosis inhibitors, Ca^2+^ channel blockers or chelators, and antioxidants to mitigate the toxic effects of CPO on erythrocytes.

## Data Availability

Data are contained within the article.
